# Gender differences of in-hospital outcomes in patients undergoing percutaneous coronary intervention in the drug-eluting stent era

**DOI:** 10.1097/MD.0000000000015557

**Published:** 2019-05-17

**Authors:** Hack-Lyoung Kim, Jae-Sik Jang, Myung-A Kim, Jae-Bin Seo, Woo-Young Chung, Sang-Hyun Kim, Seung-Jung Park, Tae-Jin Youn, Myeong-Ho Yoon, Jae-Hwan Lee, Kiyuk Chang, Myung Ho Jeong, Rak Kyeong Choi, Myeong-Ki Hong, Hyo-Soo Kim

**Affiliations:** a Division of Cardiology, Boramae Medical Center, Seoul National University College of Medicine, Seoul; b Division of Cardiology, Busan Paik Hospital, University of Inje College of Medicine, Busan; c Division of Cardiology, Asan Medical Center, University of Ulsan College of Medicine, Seoul; d Cardiovascular Center, Seoul National University Bundang Hospital and Seoul National University College of Medicine, Seongnam; e Department of Cardiology, Ajou University School of Medicine, Suwon; f Department of Cardiology, Chungnam National University School of Medicine, Chungnam National University Hospital, Daejeon; g Division of Cardiology, Seoul St. Mary's Hospital, The Catholic University of Korea, Seoul; h Department of Internal Medicine and Heart Center, Chonnam National University Hospital, Gwangju; i Division of Cardiology, Mediplex Sejong General Hospital, Incheon; j Division of Cardiology, Severance Cardiovascular Hospital, Yonsei University College of Medicine; k Department of Internal Medicine, Seoul National University Hospital, Seoul, Korea.

**Keywords:** drug-eluting stent, gender, in-hospital outcome, percutaneous coronary intervention

## Abstract

Most studies on gender difference of the in-hospital outcome of percutaneous coronary intervention (PCI) were performed in the pre-drug-eluting stents (DES) era. This study was performed to investigate whether gender influences the in-hospital outcome of PCI in the DES era.

A total of 44,967 PCI procedure between January and December of 2014 from the nationwide PCI registry database in Korea were analyzed. The study population was male predominant (70.2%). We examined the association of gender with unadjusted and adjusted in-hospital mortality and composite events of PCI, including mortality, nonfatal myocardial infarction, stent thrombosis, stroke, urgent repeat PCI and bleeding requiring transfusion.

Most of the study patients (91.3%) received DES. The incidence rates of in-hospital mortality (2.95% vs 1.99%, *P* <.001) and composite events (7.01% vs 5.48%, *P* <.001) were significantly higher in women compared to men. Unadjusted analyses showed that women had a 1.49 times higher risk of in-hospital mortality and a 1.30 times higher risk of composite events than men (*P* <.001 for each). After adjustment for potential confounders, female gender was not a risk factor for mortality (*P* = .258), but the risk of composite events remained 1.20 times higher in women than in men (*P* = .008).

Among patients undergoing PCI in the contemporary DES era, female gender was associated with an increased risk of in-hospital composite events, but not in-hospital mortality. More careful attention should be emphasized to minimize procedure-related risks and to improve prognosis in women undergoing PCI.

## Abstract 2

Supplemental Digital Content is available in the text

## 1 Introduction

Coronary artery disease (CAD) is the leading cause of death worldwide.^[1]^ Percutaneous coronary intervention (PCI) is an effective method for myocardial revascularization, and it has become the main procedure for the treatment of CAD.^[2]^ Considerable interest has been focused on the gender difference in CAD.^[3–5]^ Many studies have been performed to investigate the gender difference in in-hospital outcomes in patients undergoing PCI, but their results are still conflicting.^[6–20]^ In addition, most of these studies were performed in the era of thrombolysis, balloon angioplasty, or bare-metal stents.^[6–11,14–18]^ Although there are several recent investigations on gender issue in the drug-eluting stent (DES) era, their study population was mainly restricted to patients with acute coronary syndrome.^[12,13,19,20]^ Moreover, most studies reporting the gender issue were conducted in Western countries,^[6–13,15–20]^ and Asian data is scarce.^[14]^

Therefore, this study was performed to investigate whether there were differences in in-hospital outcomes and risk factors affecting outcomes between genders among Korean patients undergoing PCI in the contemporary DES era.

## 2 Materials and methods

### 2.1 Study population

This study data was obtained from the nationwide Korean PCI (K-PCI) registry database. The K-PCI registry database was constructed to characterize the clinical features and in-hospital PCI outcomes of Korean patients. The details of the design of the K-PCI registry and the data collection process have been previously described.^[21,22]^ Briefly, between January and December of 2014, all consecutive patients undergoing PCI were retrospectively pooled from 92 cardiac centers of Korea in this registry. The choice of medication and the types of procedural devices were left to the discretion of the operating physician. PCI was performed according to the current guidelines.^[23]^ The Korea PCI registry report was designed to construct data standards to set-up treatment guidelines reflecting relevant clinical situations and all the important aspects of coronary interventions were collected. We did not exclude certain cases for the reason of medical comorbidities, such as malignancy, renal failure or chronic liver disease. The Institutional Review Board of each participating hospital approved the study protocol.

### 2.2 Collection of clinical and angiographic data

Data was collected using web-based standardized data collection forms. A dedicated staff member at the participating hospital collected the data and forwarded it to the coordinating center where the database was created. The values of important clinical parameters were extracted from the database comprised of a standard set of 54 data elements.^[22]^ Cardiovascular risk factors were identified, which included age, hypertension, diabetes mellitus, dyslipidemia, smoking status, family history of CAD, prior myocardial infarction or PCI, chronic kidney disease, cerebrovascular disease, and peripheral arterial disease. Clinical diagnoses at the time of PCI were classified as silent ischemia, stable angina, unstable angina, and acute myocardial infarction. Left ventricular ejection fraction was measured using transthoracic echocardiography. Information on antianginal medications within 2 weeks of index PCI was obtained. These medications were beta-blockers, calcium channel blockers, long-acting nitrates, nicorandil, and trimetazidine. As angiographic parameters, extent of CAD, lesion location, PCI approach methods, and type and number of stents inserted were identified.

### 2.3 In-hospital outcomes

All-cause death, cardiac death, nonfatal myocardial infarction, stent thrombosis, stroke, urgent repeat PCI, and bleeding requiring transfusion during index hospitalization were identified. Cardiac death was defined as any death due to proximate cardiac cause (e.g., myocardial infarction, low-output failure, and fatal arrhythmia), unwitnessed death, death of unknown cause, and all procedure-related deaths.^[24]^ PCI-related myocardial infarction was considered based on clinical features including cardiac enzyme elevation between 6 and 24 hours of PCI, development of pathologic Q waves in electrocardiography or sudden unexpected cardiac death.^[25]^ Stent thrombosis was defined as definite according to the Academic Research Consortium criteria.^[26]^ Stroke was defined as a new onset of focal or global neurological deficit lasting more than 24 hours, which was confirmed by a neurologist and on brain imaging. Composite events were defined as events made up of a grouping of PCI, including mortality, non-fatal myocardial infarction, stent thrombosis, stroke, urgent repeat PCI, and bleeding requiring transfusion.

### 2.4 Statistical analysis

Continuous variables are expressed as mean ± standard deviation and categorical variables as numbers and percentages. Patient characteristics between men and women were compared using the *χ*^2^ test for categorical variables and Student *t* test for continuous variables. Unadjusted risk of in-hospital outcomes in women compared to men was assessed using the *χ*^2^ test, and adjusted risk was assessed using multiple binary logistic regression analyses. In order to identify independent risk factors for in-hospital outcomes, multiple binary logistic regression analyses were performed in men and women separately. We adjusted for clinically relevant possible confounding factors. These include demographic factors and medical history (age, hypertension, diabetes mellitus, dyslipidemia, smoking status, family history of CAD, prior history of myocardial infarction, prior history of PCI, chronic kidney disease, cerebrovascular disease, peripheral arterial disease and presentation with acute coronary syndrome), left ventricular ejection fraction, and angiographic and procedural characteristics (the extent of CAD, the number of implanted stents, and the involvement of the left main or proximal left anterior descending artery). Odds ratio (OR) and 95% confidence interval (CI) were calculated to estimate the strength of the association between risk factors and in-hospital events. All data were analyzed using IBM SPSS statistics version 24 (IBM SPSS Statistics, IBM Corp., Armonk, NY).

## 3 Results

### 3.1 Clinical characteristics of the study patients by gender

A total of 44,967 PCI procedures were analyzed in this study. Most patients (91.3%) received DES. The study population was predominant male (70.2%). Clinical, angiographic and procedural characteristics of the study patients by gender are shown in Table 1. Women were older than men (71.1 ± 10.1 years vs 62.9 ± 11.4 years, *P* <.001). Among risk factors, hypertension, diabetes mellitus, chronic kidney disease, and cerebrovascular disease were more prevalent in women than in men (*P* <.05 for each); however, current smoking, family history of CAD, previous myocardial infarction or PCI and peripheral arterial disease were more prevalent in men than in women (*P* <.05 for each). Acute myocardial infarction as a clinical presentation at the time of PCI occurred more frequently in men than in women (40.1% versus 33.3%, *P* <.001). Cardiac arrest was more frequent (2.5% vs 1.7%, *P* <.001) and left ventricular ejection fraction was lower (56.8 ± 12.0% vs 58.1 ± 12.6%, *P* <.001) in men than in women. Among antianginal medications, beta-blockers were more frequently prescribed in men and calcium channel blockers in women (*P* <.001 for each). In angiographic findings, although women were more likely to have extensive CAD, left main disease was more frequently found in men. Non-elective PCI was more frequently performed in men than in women (35.0% vs 29.5%, *P* <.001). The trans-radial approach was more frequently used in men compared to women (56.6% vs 54.8%, *P* <.001). There was no significant difference between gender in the number of stents inserted or mechanical support devices used during the procedure (*P* >.05 for each).

**Table 1:**
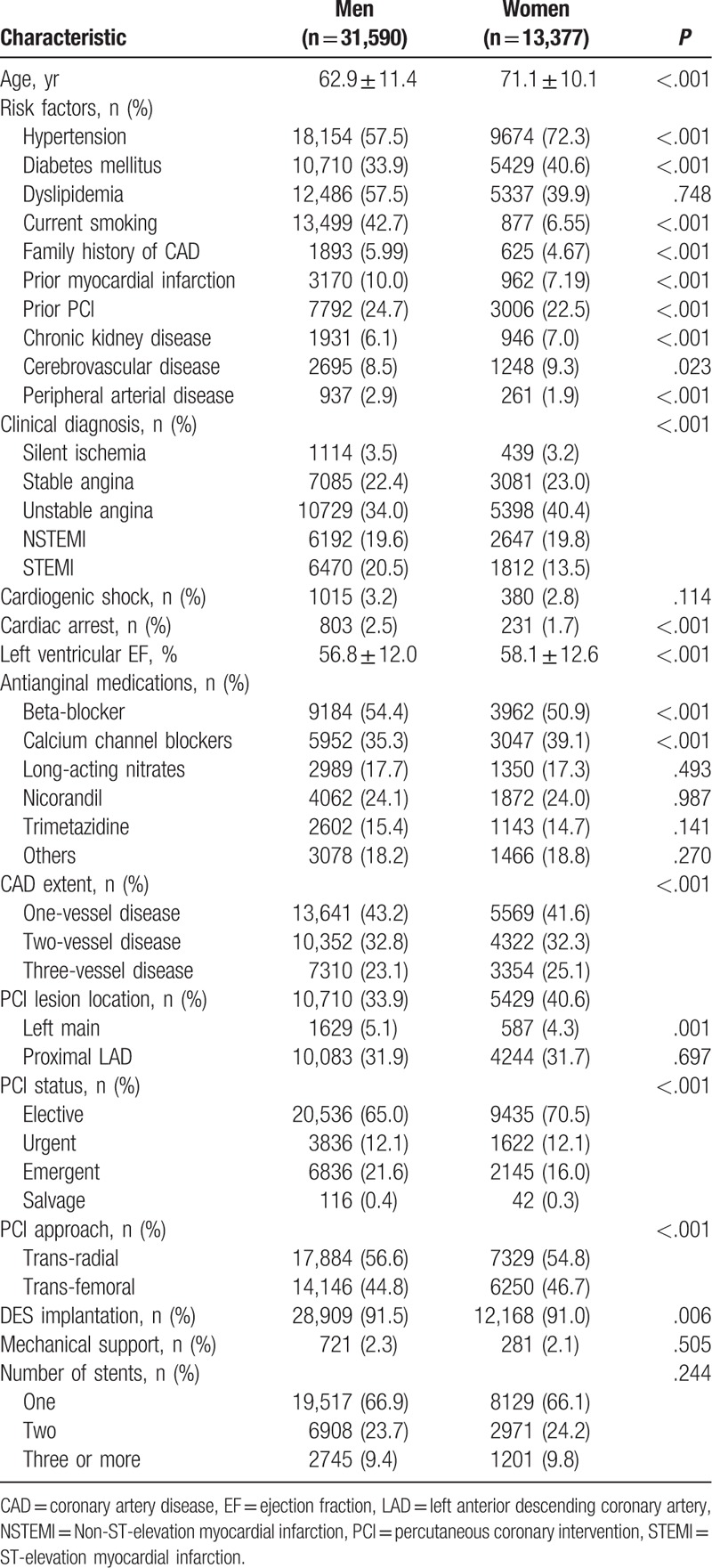
Clinical, angiographic and procedural characteristics of study patients.

### 3.2 Gender comparisons of in-hospital outcomes

**Figure 1:**
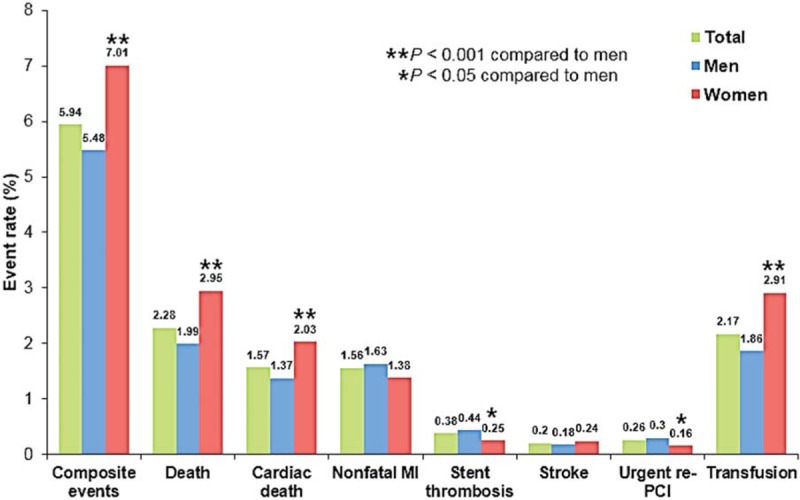
In-hospital events of PCI in men and women. MI = myocardial infarction, PCI = percutaneous coronary intervention.

In-hospital events are represented in Figure 1. There were 2669 patients (5.94%) suffering composite events during hospitalization of index PCI. The incidence of total death, cardiac death, nonfatal myocardial infarction, stent thrombosis, stroke, urgent repeat PCI and bleeding requiring transfusion were 2.28%, 1.57%, 1.56%, 0.38%, 0.20%, 0.26%, and 2.17%, respectively. The incidence of composite events was significantly higher in women than in men (7.01% vs 5.48%, *P* <.001). Total death (2.95% vs 1.99%, *P* <.001), cardiac death (2.03% vs 1.37%, *P* <.001) and bleeding requiring transfusion (2.91% vs 1.86%, *P* <.001) were more frequently occurred in women than in men; however, stent thrombosis (0.44% vs 0.25%, *P* = .003) and urgent repeat PCI (0.30% vs 0.16%, *P* = .015) more frequently occurred in men than in women. Relative risks of in-hospital outcomes in women compared to men are demonstrated in Table 2. Unadjusted analyses showed that women had a 1.49 times higher risk of in-hospital mortality (95% CI, 1.31–1.69; *P* <.001) and a 1.30 times higher risk of composite events (95% CI, 1.19–1.41; *P* <.001) than men. After adjustment for potential confounders, female gender was not a risk factor for mortality (OR, 1.25; 95% CI, 0.84–1.86; *P* = .258), but it remained as a significant predictor for composite events (OR, 1.20; 95% CI, 1.05–1.37; *P* = .008).

**Table 2:**
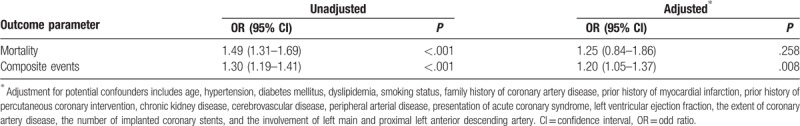
Women's risk for in-hospital outcomes compared to men (n = 44,967).

In subgroup analysis, in-hospital composite event rates were similar between genders in younger age groups (<55 years) (*P* = .417). However, in-hospital composite event rates were significantly higher in women than in men in older age group (≥55 years) (*P* <.001). Event rates in women were significantly higher whether they had diabetes mellitus or presented with acute myocardial infarction (*P* <.001 for each) (Supplementary Table).

### 3.3 Independent risk factors for the in-hospital outcomes in men and women

Independent risk factors associated with in-hospital outcomes in men and women are separately shown in Table 3. In men, age, dyslipidemia, previous history of PCI, chronic kidney disease, peripheral arterial disease, acute coronary syndrome, extent of CAD, and involvement of the left main coronary artery or proximal left anterior descending artery were independently associated with in-hospital outcomes in the multivariable analysis. In women, old age, diabetes mellitus, family history of CAD, chronic kidney disease, acute coronary syndrome, lower left ventricular ejection fraction, and extent of CAD and involvement of the left main coronary artery were independent predictors of in-hospital outcomes. Old age, chronic kidney disease, clinical presentation with acute coronary syndrome, left ventricular systolic dysfunction, more severe CAD and left main disease were common risk factors in both men and women. However, some other risk factors showed gender differences: dyslipidemia, prior history of PCI, peripheral arterial disease, and the lesion of the proximal left anterior descending coronary artery were independent risk factors in men but not in women; however, diabetes mellitus and family history of CAD were independent risk factors in women, but not in men.

**Table 3:**
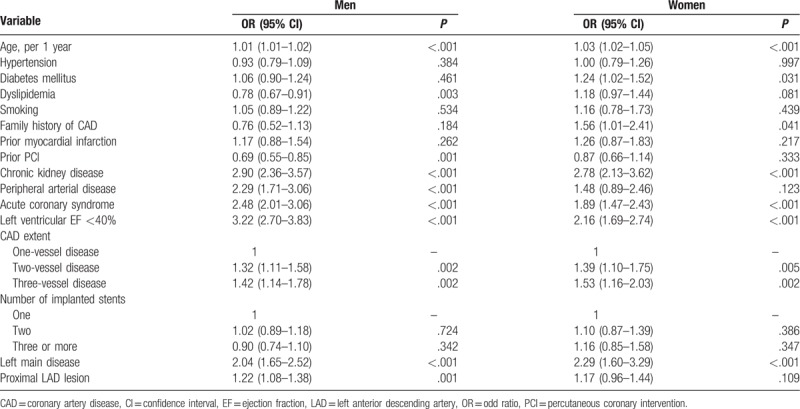
Independent predictors of composite in-hospital events in men and women.

## 4 Discussion

The gender difference in PCI-related in-hospital outcomes has not been well addressed in the DES era especially in Asian patients. Using the nation-wide registry data of Korean patients undergoing PCI with DES, this study showed that women had higher in-hospital composite events than men. However, in-hospital mortality rates were not different between genders.

There have been several studies demonstrating gender differences in in-hospital outcomes in patients undergoing PCI. Although some studies have reported higher event rates in women than in men,^[6–13]^ several studies have failed to show gender differences because gender differences in in-hospital outcomes decreased or disappeared after adjustment for age, comorbidity, treatment, and procedure.^[14–20]^ Our study also demonstrated that in unadjusted analyses, the risk of in-hospital mortality in women disappeared after controlling for baseline differences in age, comorbidities, disease severity, and angiographic and procedural characteristics. Some studies showed that female gender did not predict in-hospital mortality independently, but remained an independent risk factor for PCI complications,^[6,27]^ which is in line with our results showing that female gender was independently associated with increased in-hospital composite outcomes including bleeding requiring transfusion but not in-hospital mortality. It has been suggested that a high relative risk in women has gradually decreased as the practice of PCI has evolved over the last decades.^[10,18]^

Most studies reporting on the outcomes of PCI for women versus men were conducted in the thrombolytic reperfusion era, the balloon angioplasty era or the bare-metal stent era, before the widespread availability of DES,^[6–11,14–18]^ but a greater proportion of PCI procedures are currently being performed using DES. Moreover, PCI tools such as guiding catheters, wires and balloon catheters have been developed, and more effective and safe adjunctive pharmacological therapies have been developed. However, there have been limited data on the effect of gender on in-hospital outcomes in the DES era. Although there are several recent investigations on gender issue in the DES era, their study populations were restricted to patients with acute coronary syndrome, and their results are still conflicting.^[12,13,19,20]^ Therefore, results of our study may deserve clinical attention, because we used the recent database of PCI registry reflecting the current practice of interventional cardiology among unselected patients with CAD. In addition, most of the previous studies were performed in Western countries,^[6–13,15–20]^ and the gender issue on PCI outcome among Asian patients remained to be evaluated. From this point of view, our study performed on Korean patients has another strength.

We showed a higher in-hospital composite event rates in women than in men. Several characteristics showing sex disparity including advanced age, more risk factors such as hypertension and diabetes, more severe CAD extent, delayed PCI procedure, and transfemoral approach, can be considered possible causes explaining a higher event rate in women. It has been shown that there is a 10- to 20-year delay in the onset of CAD in women when compared to men.^[28,29]^ Advanced age in women carries more cardiovascular risks and comorbidity, leading to less frequent use of invasive treatment.^[6,11,17,29]^ Our study suggests that advanced age with increased risk profile of women may be the main mechanism of gender gap because the gender difference of in-hospital composite events narrowed and in-hospital mortality disappeared after adjustment for these risk factors. In addition, atypical presentations in women may delay hospital visit and revascularization therapy.^[4,5]^ It could be also postulated that smaller artery size^[6,13,19,30,31]^ and more frequent use of femoral artery access^[32]^ in women are associated with increased periprocedural vascular complications or bleeding. Our study showed a consistent finding in that there was a significantly higher rate of bleeding requiring transfusion in women compared to men Bleeding avoidance strategy such as preferred selection of radial artery access, the use of closure device and avoidance of inappropriate use of glycoprotein inhibitor should be considered in especially in women.^[33]^ In addition, intensive monitoring and aggressive management for bleeding complication after PCI should be applied to women in order to improve their in-hospital outcome.

We found independent risk factors of in-hospital outcomes in men and women with separate multivariable analyses. Advanced age, chronic kidney disease, acute coronary syndrome, left ventricular systolic function <40%, involvement of 2 or more vessels, and left main disease were independent risk factors for in-hospital composite events in both men and women. These are also well-known periprocedural risk factors of PCI in many previous studies.^[15,17,18,30]^ Interestingly, there were gender differences in some risk factors for in-hospital outcomes in our study. Multivariable analyses controlling potential confounders showed that presence of peripheral arterial disease and involvement of the proximal left anterior descending artery were risk factors, while history of prior PCI and dyslipidemia were protective factors in men, but these variables were not independent predictors of in-hospital outcomes in women. Whereas, presence of diabetes mellitus and family history of CAD were risk factors in women but not in men. Consistent with our finding, it has been reported that the excess risk of cardiovascular events associated with diabetes is significantly higher in women than in men.^[34,35]^ It is important to recognize gender differences in risk factors because this might result in a better understanding of gender-related mechanism of CAD, and improved therapeutic strategies and outcomes in both men and women. Other risk factors showing gender disparity should be further validated in additional studies.

It has been suggested that clinical studies in recent decades have not always adequately enrolled women or analyzed gender differences in the data.^[36]^ This problem has been an obstacle for the progression of understanding women's clinical characteristics. Considering that women had significantly higher vascular complication rates as shown in our and other studies, the gender issue should be considered and more careful attention should be paid to women in order to minimize procedure-related vascular complications. We also showed different independent risk factors for in-hospital outcomes between men and women, which may provide more detailed information on high-risk patients. These high-risk patients need more aggressive management and monitoring, and specific measures aimed at preventing periprocedural events in this group of patients may improve in-hospital prognosis.

There are several limitations in this study. First, as our results are obtained from a retrospective analysis of an observational PCI registry that was subject to missing or incomplete information. Well-controlled prospective trials are required to confirm our findings. However, this registry can provide “real-world” data on a wide spectrum of unselected patients that underwent PCI procedures in Korea. Second, our study focused on in-hospital outcomes, and outcomes information after discharge was not available. Third, not all variables were controlled in the multivariable analyses, and there may remain significant unrecognized differences between men and women. Specifically, data on the use of potent antiplatelet agents such as glycoprotein IIb/IIIa inhibitor or ticarglrelor was not available in our study. These medications may have impacted on periprocedural ischemic and bleeding complications. Finally, information on menopausal status and hormone replacement therapy was not available in the present study. This information may be valuable to understand underlying pathophysiology of gender difference.

## 5 Conclusion

In this nation-wide registry of contemporary PCI in Korea, women showed a higher in-hospital composite event rates associated PCI with DES than men. However, female gender was not an independent predictor for in-hospital mortality when adjusted for important clinical covariates. More careful attention to women should be emphasized to minimize procedure-related risks and improve prognosis.

## Author contributions

**Conceptualization:** Hack-Lyoung Kim, Myung-A Kim.

**Data curation:** Jae-Bin Seo, Woo-Young Chung, Sang-Hyun Kim.

**Formal analysis:** Jae-Sik Jang, Sang-Hyun Kim.

**Methodology:** Jae-Bin Seo, Woo-Young Chung.

**Resources:** Seung-Jung Park, Tae-Jin Youn, Myeong-Ho Yoon, Jae-Hwan Lee, Kiyuk Chang, Myung Ho Jeong, Rak Kyeong Choi, Myeong-Ki Hong, Hyo-Soo Kim.

**Supervision:** Myung-A Kim.

**Validation:** Woo-Young Chung.

**Visualization:** Sang-Hyun Kim.

**Writing – original draft:** Hack-Lyoung Kim, Jae-Sik Jang, Myung-A Kim.

**Writing – review & editing:** Hack-Lyoung Kim, Jae-Bin Seo, Seung-Jung Park, Tae-Jin Youn, Myeong-Ho Yoon, Jae-Hwan Lee, Kiyuk Chang, Myung Ho Jeong, Rak Kyeong Choi, Myeong-Ki Hong, Hyo-Soo Kim.

## Supplementary Material

Supplemental Digital Content
