# Supplementary material for: Gender differences of in-hospital outcomes in patients undergoing percutaneous coronary intervention in the drug-eluting stent era
Source: Medicine (Baltimore). 2019 May 17;98(20):e15557. doi: 10.1097/MD.0000000000015557 (PMC6531253; doi:10.1097/MD.0000000000015557)
Supplement: Supplemental Digital Content [file medi-98-e15557-s001.doc]

**Supplementary Table. Gender difference of in-hospital composite event rates according to age, diabetes and acute myocardial infarction**

| **Subgroup** | **Men**  **(n = 31,590)** | **Women**  **(n = 13,377)** | ***P*** |
| --- | --- | --- | --- |
| *Age* |  |  |  |
| < 55 years (n = 8,541) | 309 (4.1) | 43 (4.7) | 0.417 |
| ≥ 55 years (n = 36,426) | 1,422 (5.9) | 895 (7.2) | < 0.001 |
| *Diabetes mellitus* |  |  |  |
| No (n = 28,803) | 1,036 (5.0) | 498 (6.3) | < 0.001 |
| Yes (n = 16,139) | 685 (6.4) | 435 (8.0) | < 0.001 |
| *Acute myocardial infarction* |  |  |  |
| No (n = 27,846) | 425 (2.2) | 284 (3.2) | < 0.001 |
| Yes (n = 17,121) | 1,306 (10.3) | 654 (14.7) | < 0.001 |

Numbers are presented as n (%).
